# Casting light on the workings of illegal fishing markets

**DOI:** 10.1073/pnas.2512081123

**Published:** 2026-08-10

**Authors:** Philippe Le Billon, Zelda Ladefoged, U. Rashid Sumaila

**Affiliations:** ^a^ Department of Geography and School of Public Policy and Global Affairs, University of British Columbia, Vancouver, BC V6T 1Z2, Canada; ^b^ Department of Geography, University of British Columbia, Vancouver, BC V6T 1Z2, Canada; ^c^ Institute for the Oceans and Fisheries and School of Public Policy and Global Affairs, University of British Columbia, Vancouver, BC V6T 1Z2, Canada

**Keywords:** illegal fishing, illegal markets, IUU, ocean governance, seafood

## Abstract

About 3.5 million fishing vessels ply the world’s oceans, providing livelihoods and food for millions of people. Sound management is key to fisheries’ sustainability, but illegal fishing, along with fishing overcapacity generally and environmental change, are putting many fisheries at risk. Illegal fishing is a globally pervasive yet unevenly distributed phenomenon, manifesting in diverse forms across both small-scale and industrial-scale fleets, the latter accounting for about 82 to 93% of the 8.4 to 15.4 million metric tons global annual illicit marine wild fish seafood trade valued at US$6.2 to 12.2 billion. The massive rise in fishing effort since 1950 has not been matched by adequate regulations and enforcement, especially for industrial-scale fishing in the waters of developing countries. About two-thirds of the illicit trade by value originates from fishing grounds in West Africa (27%), East Asia (24%), and Southeast Asia (16%). Remote sensing and AI technologies are improving detection of illegal fishing, but enforcement remains an issue given its high costs and the relative ease of laundering illegal catch through transshipments at sea, poorly regulated ports, and fraudulent documentation along seafood supply chains. Illegal fishing markets require multilayered policy responses grounded in transparency, international cooperation, and socioeconomic justice. Proposed measures, ranging from mandatory electronic vessel identification and subsidy reform to artisanal fishery protections and stronger port controls, warrant further research into their implementation and effectiveness.

Illegal fishing, meaning the violation of local or international regulations, is a widespread and opportunistic phenomenon, particularly affecting areas with enforcement challenges (1) or weak governance (2–4). Documented across many societies since at least Roman times (5, 6), concerns over illegal fishing increased with the acceleration of industrial-scale fishing activities, depletion of fish stocks, threats to traditional coastal livelihoods and food security, and links with organized crime and national security issues (4, 7). Illegal fishing undermines marine conservation and fisheries management efforts (8) and can fuel corruption (9), weaken governance, and involve grave human rights abuses (10). Illegal fishing is particularly damaging compared to legal fishing when carried out by industrial vessels using destructive gear (11), targeting overexploited and poorly managed stocks (2), operating in near-shore breeding grounds vital to artisanal fishers and future fish populations (12), undercutting the livelihoods and public revenues of fishing-dependent local communities, and exposing crews to abuse with little protection from their home governments (13).

Offenders primarily engage in illegal fishing and associated violations, such as fraud and forced labor, because the potential benefits of breaking rules are perceived to outweigh the perceived risk and consequences of getting caught (14–16). These violations often occur in fisheries challenged by fleet overcapacity, ineffective management, and harmful subsidies undermining sustainability and fair competition within the seafood sector (15, 17), such as subsidies for fuel, vessel construction or modernization, and access to distant fishing grounds (17). These can incentivize unsustainable practices, undermining fish stock recovery through overfishing, overcapacity, and illegal fishing. Additional drivers of illegal fishing, especially among small-scale fishers, include moral and other social considerations (e.g., personal values, standing within society, acceptability of illegal practices within fishing communities) (1), as well as disenfranchisement from fisheries and maritime access, opposition to regulations that are viewed as illegitimate or restrictive, exclusionary conservation policies, and economic hardship (14, 18–20). In turn, unchecked illegal fishing economically deprives legitimate fishers and exacerbates inequities (21, 22), with competition over dwindling resources driving more fishers toward illegal practices or alternative options such as smuggling or outmigration (23). Besides illegal fishing per se, companies often abuse workers’ rights to reduce labor costs, register vessels under Flags of Convenience (FoC) to unfairly reduce regulatory costs, resort to fraud to cover their tracks and inflate profits, illegally shelter profits from taxation through transfer pricing between undeclared affiliates, and set up complex corporate structures to limit liabilities and hide ultimate beneficial ownership (24). Industrial fishing companies that are reported as being among the main perpetrators of illegal fishing have also been associated with other forms of financial crimes such as defrauding of shareholders through inflated assets (25). Illegality can also result from and exacerbate tensions over disputed maritime areas (26–28). In such cases, assertions of sovereignty may be one of the prime motivations for fishing illegality, as seen in disputed South China Sea areas where states have put “fishers and fishing regulation on the frontlines of defending their territorial claims,” in effect instrumenting the fishing sector to pursue broader geopolitical claims (29).

Illegal fishing’s paradigmatic products include internationally traded high-value seafood, such as abalone, sea cucumbers, salmon, shark fins, shrimps, toothfish, and tuna (2, 4, 30). However, illegal fishing affects many species, either directly or indirectly through bycatch or harmful gear damaging ecosystems and increasing unreported discards (31, 32). In depleted fisheries, illegal fishing may turn to juveniles or low-value species key to local food security and fisheries sustainability (33). Product differentiation is important as species require specific fishing practices, equipment, trading networks, and markets. Sharks, for example, can be caught in coastal waters and their high-value fins dried in the sun, making it possible for even small-scale fishing in remote locations to partake in international markets for illegal products (34, 35). Some species, such as tuna or white fish, are commonly traded and therefore more easily laundered through mixing with legal catch (36), whereas others such as the totoaba, a giant croaker endemic to the Sea of Cortez that is fished for its swim blader, fall under more stringent domestic or international protection and therefore need to be smuggled through criminal networks often involving corrupt officials (37, 38). Services tied to illegal fishing, such as fake documentation and unlawful transshipment of fish at sea, allow rule-breakers to cut costs and outcompete the rule-followers (4, 32, 39).

Accounting for an estimated 8 to 15% of global world marine wild capture catch between 2015 to 2019, the illicit trade in marine catch harms ecosystems, undermines the sound management of fish stocks, and can cause significant food insecurity and economic losses for communities and countries relying on fishing industries (4), especially for island states or low-income coastal states with limited alternatives (40). It can also contribute to other illegal markets, such as smuggling and human trafficking (25, 41), increase the likelihood of piracy (42), and become a major dimension of international maritime disputes (43). While rapid technological progress is improving the detection of illegal fishing, shifting economic and moral incentives toward legal practices, as well as designing regulations and implementing comanagement that protect vulnerable traditional coastal communities and their fishing grounds, are key to promoting just and sustainable fisheries in the long-term.

## 1. Defining Illegal Fishing

Fishing illegality is grounded in international and domestic laws regulating marine resource use and property rights (7, 44). It includes the violation of fishing regulations, maritime jurisdiction boundaries, and laws designed to “conserve and manage fish stocks” for the purpose of protecting social and economic opportunities, food security, and/or ecosystems (45). Illegal fishing comes under the umbrella term of “illegal, unreported, and unregulated” (IUU) fishing (45). “Illegal” refers to activities by either foreign or domestic vessels occurring within waters governed by individual countries or Regional Fisheries Management Organizations (RFMO) and directly violating their authority and regulatory frameworks. “Unreported” refers to activities that are misreported or not reported to relevant authorities. “Unregulated” refers to activities involving vessels without nationality or those flagged to nonmember States operating in areas or targeting species under a RFMO’s mandate, as well as fishing for stocks without management regulations, in a manner inconsistent with States’ obligations to conserve living resources on the high seas (UNCLOS art. 117). The term IUU thus covers a broad spectrum of activities and regulatory contexts (46, 47). Here, we focus on industrial-scale illegal fishing, including illegally unreported catch, which accounts for about 85% of global illicit marine fish trade, compared to recreational, artisanal, or small-scale fishing, which primarily involves individual households using relatively small vessels (typically under 12 m) for nearshore trips, or no vessel at all when fishing from shore, for mainly local consumption (48, 49).

Illegal fishing practices often arise following periods of legal overfishing and fisheries mismanagement (50, 51), but colonialism, racism, and the drive to “modernize” fisheries and privilege industrial-scale fishing have been prime, if unstated, factors in many fisheries policies and anti-IUU enforcement (22, 52). It is thus important to distinguish between profit-motivated illegal practices undertaken with deliberate criminal intent, more characteristic of industrial fishing, and those arising from informal economies, social marginalization, poverty, or customary practices of small-scale fishers. Many traditional fishing grounds and tenure systems have been effectively “illegalized” with the introduction of new regulations like the Law of the Sea’s Exclusive Economic Zones (EEZ), along with marine protected areas, and fisheries management regimes like area-based quotas (22, 53). These exclusions and restrictive rules can put fishers in situations that are now classified as illegal or unreported, forcing them to either change or abandon their long-standing practices or operate in violation of the new rules (22, 53). In turn, a culture of noncompliance is fostered when fishers see regulations as illegitimate, such as those contradicting customary fishing practices or excluding Indigenous fishers (54). Grievances over regulatory burdens and disproportionate penalties more heavily punishing small-scale fishers can exacerbate fishing conflicts and illegal practices, which in turn can disrupt social status patterns in fishing communities and incentivize noncompliance through shifts in self-interest, perceptions, and social norms (54, 55). Grievances, debt, and social pressure can also drive fishers into other illegal activities, including smuggling.

## 2. Extent of Regulation and Enforcement

Several major international instruments seek to regulate IUU fishing (56). The UN Convention on the Law of the Sea (UNCLOS) requires states to protect the marine environment, empowers coastal states to prosecute fishing activities within their EEZ, and enables Flag states to enforce regulations for their registered vessels operating on the high seas. With 84 parties, the international Port State Measures Agreement to Prevent, Deter and Eliminate IUU (PSMA) mandates and empowers parties to prevent foreign vessels implicated in IUU fishing from utilizing their ports and to take punitive measures against offenders (57). The International Plan of Action to Prevent, Deter and Eliminate Illegal, Unreported and Unregulated Fishing offers a comprehensive toolbox for Flag, coastal, and market states as well as RFMOs, to develop and enforce anti-IUU measures. In addition, several other international instruments indirectly address IUU fishing, including the UN Fish Stocks Agreements, the Cape Town Agreement that sets safety standards for fishing vessels, and the Convention for the Prevention of Pollution from Ships that regulates environmental impacts.

Illegal fishing persists largely because gain opportunities far exceed the risk of penalties. The industry’s unique spatial and structural characteristics (large economic area policed with relatively small monitoring/enforcement budgets) means that the distribution of costs and benefits heavily favors offenders compared to regulators/authorities. The probability of paying a penalty for IUU fishing is often extremely low, as it depends on the likelihood of boarding, detection, and successful prosecution (58). Sumaila et al.’s 2006 study showed that even with a 20% detection rate, contemporaneous fines needed to increase 24-fold to make IUU fishing uneconomic and/or unprofitable (1). At a more realistic/conservative detection rate (5 to 10%), fines would need to be 74 to 173 times higher (1). Consequently, operators often treat fines as operational costs and continue to fish illegally.

Even when offenses are reported, beneficial owners can avoid penalties as a result of convoluted ownership networks (59, 60) and by changing vessel names and flags (1), allowing them to effectively disappear as chasing suspected offenders is logistically and legally complex for many regulatory bodies. Beyond increasing detection rates, penalties, and international cooperation, making illegal operations costlier by mechanisms such as by pushing illegal fishers to sail a long way to find a corrupt port for landings is part of the solution. So are economic and moral incentives for IUU fishers to move toward legal practices, including lower access fees, preferential market access, cheaper insurance, as well as social norms and institutional recognition (54, 61).

Many case studies (61–63) suggest that fisheries management works best when it is built on collaboration rather than coercion. A whole-of-society approach to fisheries sustainability (including education), formal and informal incentives for compliance, structures favoring “prosocial” behavior (e.g., small cooperatives), and the promotion of alternative livelihoods can complement or outperform top–down repressive approaches (62–66).

## 3. Fishy Markets

Aquatic products are among the most internationally traded food commodities in the world (67). In 2022, global production of aquatic animals reached a record high of 185 million metric tons (MMT) valued at $452 billion. Through to the mid-20th century, aquatic production was dominated by wild fish, delivering 19 MMT in 1950 compared to aquaculture’s 0.64 MMT (68). However, since the late 1980s catch of wild fish has stagnated despite increased fishing effort, accounting for only 49% (91 MMT valued at $157 billion) of total production in 2022 (69). Although difficult to assess, IUU fishing possibly peaked in the late 1990s to 2000s. Between 2000 and 2003, illegal and unreported fishing was estimated at 11 to 26 MMT annually, with a mean of 18% (13 to 31%) of total marine global catch (2). Based on assumed diversion from unreported catch identified through catch reconstruction (70), illicit trade in seafood would range from 7.7 to 14 MMT per year with a landed value of $8.9 to 17.2 billion between 2005 and 2014 (70) and from 8.4 to 15.4 MMT per year valued at $6.2 to 12.2 billion between 2015 and 2019 (*SI Appendix*).

**Fig. 1.**
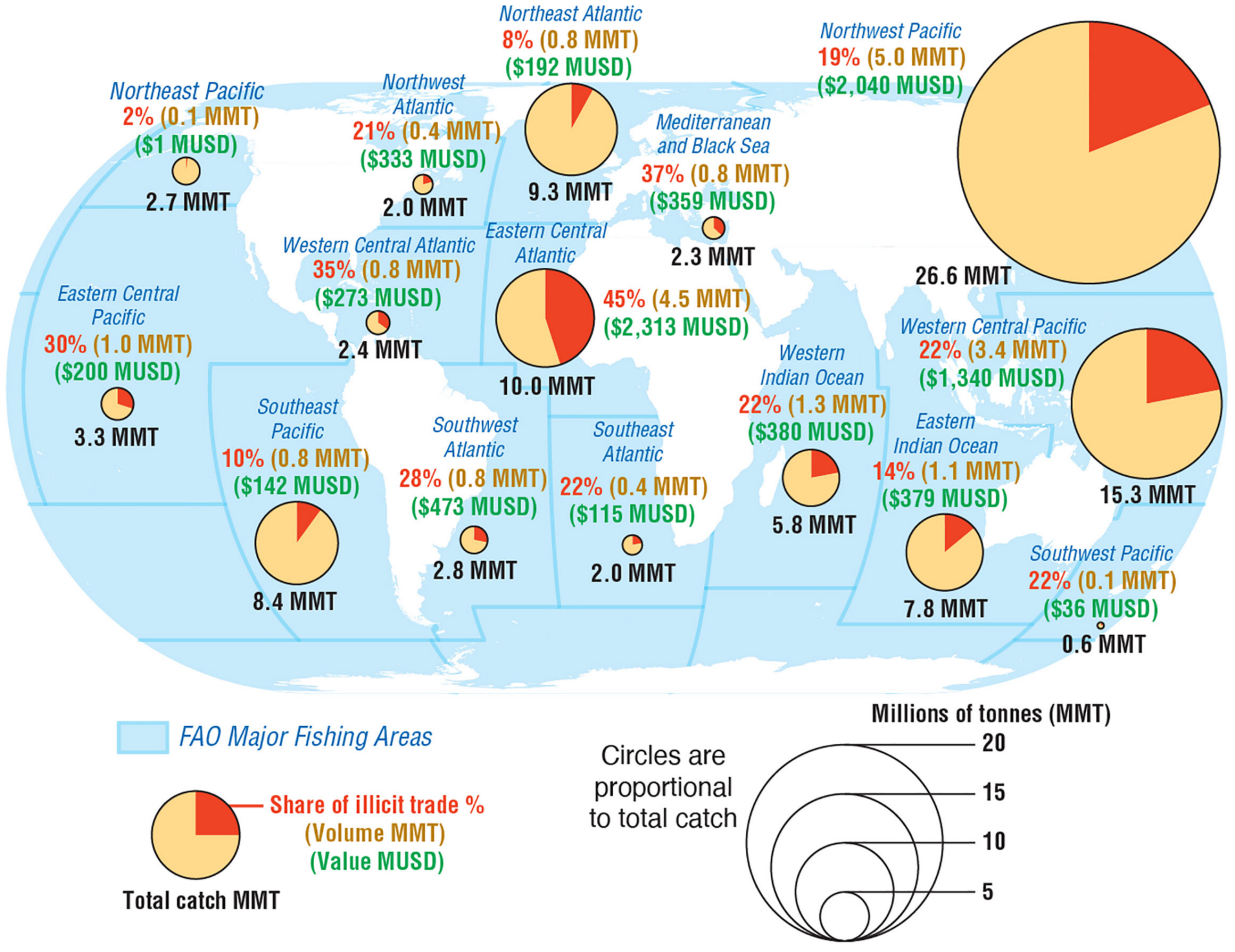
Volume of total catch (circle) and share (pie and number in red), volume (brown), and value (green) of illicit trade by FAO area, 2015 to 2019 (81).

Illegal fishing and its impacts are unevenly distributed, with Asia, Africa, and South America collectively accounting for approximately 80% of global IUU catch and 82% of revenue losses for 2005 to 2014 (70–74). An updated analysis based on United Nations Food and Agriculture Organization (FAO) fishing areas for 2015 to 2019 data point to a similar pattern (Fig. 1), with about two-thirds of the illicit trade in value originating from the Atlantic Eastern Central/West Africa (27%), Pacific Northwest/East Asia (24%), and Pacific Western Central/Southeast Asia (16%). If illegal fishing tends to concentrate in the waters of low- and lower-middle income countries, the products derived are mostly consumed in upper-middle and high-income countries. In 2007, $1.5 billion of the US$23.5 billion in extra-EU seafood product imported into the European Union (EU) was linked to IUU fishing (75). Similarly, US seafood imports totaled US$32 billion, with US$2.4 billion from IUU sources in 2019 (76). For Japan, IUU were estimated at 24 to 36% of the 2.15 MMT, or 12 to 18% of the $13 billion in wild-seafood imports (77). China imported $22.5 billion in seafood, but no estimates are yet available (77). This not only reflects the globalized nature of seafood trade but also the influence of Distant Water Fishing (DWF) fleets exploiting weak governance and enforcement in developing countries’ waters and the high seas. The top five DWF fleets (China, Taiwan, Japan, South Korea, and Spain) account for nearly 90% of global DWF efforts, with China and Taiwan responsible for 38% and 21.5%, respectively, between 2015 and 2017 (78). These fleets concentrate their efforts in the Pacific, East Africa, and West Africa (79). DWF operations, driven by economic incentives like subsidies and high-value fish markets, are disproportionately located in regions with poor enforcement capacity and limited governance (80). By exploiting these vulnerabilities, DWF fleets concentrate illegal fishing activities, transforming regions of low governance into hotspots of illicit activity (80). Three of the top 10 companies with the highest fishing effort in the high seas were also among the top 20 companies with the highest number of offenses (25).

## 4. Illegal Seafood Supply Chains

Illegal seafood supply chains range from direct sales to consumers by artisanal fishers to long transnational supply chains involving multiple corporations (82). At the industrial scale, supply chains start with vessels violating fishing regulations and laundering their illegal catch through at-sea transshipments onto refrigerated cargo ships (“reefers” or “carriers”), ports with lax enforcement, or legitimate ports after fraudulent laundering operations before generally being sold through local domestic markets or shipped via hard-to-inspect refrigerated containers (70, 83). At-sea transshipments of illegal catch allow fishing vessels to bypass port inspections for months or even years while continuing to fish and maintain workers under exploitative conditions (84). Fishing vessels suspected of illegal fishing are more likely to visit ports with more stringent inspections after meeting with carriers, suggesting that any suspicious catch has already been transferred before docking (10). Conversely, high-risk carriers under FoC tend to visit ports that have low monitoring, control, and surveillance capacity and are often Chinese-affiliated (85). This lack of oversight persists even in wealthy fisheries such as the tuna sector managed by the Western and Central Pacific Fisheries Commission, which is valued at $22 billion annually and regarded as one of the world’s most intensively regulated and monitored transboundary fisheries. In this case, the vast majority of transshipments could not be independently verified using transparent and publicly accessible data (86).

**Fig. 2.**
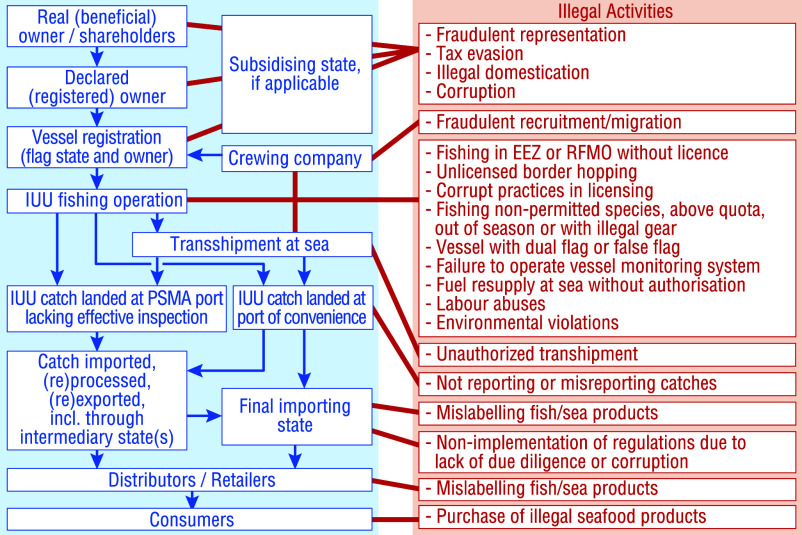
Fishing supply chain (blue) and illegal activities (red) (89).

Internationally traded illegally caught fish are distributed through complex networks that often involve fraud, document falsification, bribery, and blending with legal catches, see Fig. 2 (87). Illegal catch is generally laundered through legitimate markets. For example, it is mixed with legal catch (either aboard or on reefers), catch reports are falsified, and fish batches are landed in multiple ports to stay within quota limits. Down the chain, mislabeling and other processing, packaging, labeling, shipping, and retail-related fraud allows IUU origin products to masquerade as legitimate in retail markets and restaurants. Some IUU-caught fish species are distributed through parallel markets, either through short local circuits often using personal connections (e.g., species caught above quotas), or more elaborate long-distance smuggling networks (e.g., species internationally protected under the Convention on International Trade in Endangered Species) with sometimes pre-export processing to reduce detection (37, 88).

Illegalities can happen in different ways along the production and distribution process, including illegal fishing itself (use of prohibited gear or lack of license); “high grading” by discarding fish of lesser value and replacing it with higher value fish to maximize revenue and profits from fixed quotas (bottom trawlers targeting shrimp are notorious for this) (90); landing more fish than declared by fisher and buyer/processor so that either the fisher remains within quotas while earning more revenues or the buyer gets more fish than what they officially pay for; selling whole round fish weight while reporting much lighter gutted and headed fish; falsely declaring a specie of lower value or higher volume of quotas, rather than the one actually landed; using forced labor in fishing or processing (e.g., Chinese seafood processors using Uyghur and North Korean workers) (91, 92); and fraudulently labelling a specie of lower value than the one sold and other forms of adulteration (93, 94).

## 5. Shadow Fleets and Corporate Networks

By 2022, the global maritime fishing fleet was estimated at about 3.5 million vessels, with around half a million between 12 to 24 m in length (69) and only 64,000 that were over 24 m (63). The relatively small number of large ships accounts for a disproportionate share of the marine catch—both legal and illegal. Based on synthetic-aperture radar detection, there was on average at least 28,800 fishing vessels above 15 m out at sea at any point in time during 2017 to 2021, with only about one-quarter publicly visible via their electronic identification systems. Although the intentional or unintentional absence of electronic identification signal does not automatically imply that the fishing vessels are engaged in illegal fishing activities, this finding potentially suggests a much higher level of IUU activities than previously estimated—at least, in regions such as West Africa and Southeast Asia (95). The size of individual enterprises involved in illegal fishing varies widely, from companies with a single fishing boat to large integrated companies with hundreds of vessels. On average, companies with IUU fishing offenses owned, on average, 5.74 fishing vessels with a total Gross Registered Tonnage of 6,348 tons (96), but links between companies and ultimate beneficial ownership are frequently concealed by complex corporate structures (24, 97). Transshipment vessels are mostly controlled by companies from Russia (26%) and China (20%), although the individual company with the largest number of such vessels (N = 46) is Greek (98).

There is no global data on the total number of fishing enterprises and the industrial fishing sector is relatively concentrated than most other ocean economic sectors (99), but there were about 13 corporations “controlling 11 to 16% of the global marine catch (9 to 13 million tons) and 19 to 40% of the largest and most valuable stocks” in 2012 (100). In 2020, the ten largest seafood companies reported annual revenues of between US$ 1 to 11 billion (101). Large industrial-scale fishing companies are often vertically integrated from fishing to processing, though retail distribution is generally separate. Integrated supply chains can facilitate traceability and expose sourcing from illegal fishing (102), but they can also help internally conceal offenses and launder illegal catch, especially if transshipments mix of the large legal and illegal catches and landing takes place through a company’s own infrastructure. Outside of the large integrated companies, supply chains can involve many players, including aggregators and traders, primary and secondary processors, as well as wholesalers, distributors, and transporters. Longer supply chains complicate traceability and increase fraud risks as each player seeks to cut costs and increase profits.

China has become the world’s largest fishing country, with its catch increasing from 2.7 MMT in 1980 to a peak of 17 MMT in 2014, with about 11.8 MMT out of the estimated global catch of 79.7 MMT in 2022) (69). It also dominates fish processing and export markets with 6.2 MMT in 2020, including 3.1 MMT of re-exported catches from foreign countries (69, 103). A third of the 972 industrial and semi-industrial vessels listed or formerly listed for IUU fishing between 2010 and 2022 by the nonprofit fisheries intelligence organization TMT, were flagged to China (41). Up to 16,966 Chinese vessels are fishing outside of the country’s EEZ, including between 1,989 and 3,432 vessels operating outside neighboring EEZs, making China’s by far the world’s largest DWF fleet (104). Among the 20 Chinese DWF companies with the highest number of reported IUU-fishing offenses, 45% had between 2 to 18 vessels, 45% had between 24 to 59 vessels, and two companies had 188 and 429 vessels, respectively (105).

Enterprises involved in recurrent industrial-scale IUU fishing are generally durable legal businesses, yet often registered in tax havens and with an opaque structure shaped around family ties and business associates that operate discreet subsidiaries owning vessels under different names and flags (“flag-hopping”) (25, 41). Opaque joint-ventures between foreign investors and domestic elites allow for the domestication of foreign vessels outcompeting local fishers (106). With a growing number of Chinese fishing vessels illegally entering its EEZ after the 1980s, Ghana instituted laws to restrict fishing licenses to domestic companies and prohibit foreign ownership of Ghana-flagged trawlers. Yet, many trawlers now operating in Ghana are illegally beneficially owned by Chinese companies (107), resulting in annual losses of US $14.4 to 23.7 million in license fees and enforcement penalties (107). Local crew have described the Ghana Industrial Trawlers Association as protecting the interest of Ghanaian-owned front companies hiding Chinese beneficial owners, in effect creating a cartel of local politicians and foreign fishing companies maintaining an exploitative system harming small-scale fishers and coastal communities (108), and benefiting powerful local entrepreneurs, corrupt politicians, and Chinese owners (24, 97).

Boats generally represent the main capital investment for IUU fishing, yet cheap vessels are also available on the used market and “the profits of a ship from a single voyage may significantly exceed the price of the ship itself” (4). For highly profitable IUU fishing, such as Patagonian toothfish in the Southern Ocean in the late 1990s, operators simply abandoned vessels when caught and purchased new ones, as earnings usually outweigh vessel costs (109). Low boat prices can result from government subsidies for vessel buybacks or fraudulent decommissioning (e.g., boats returning to sea rather than being destroyed) (110, 111). Industrial fleets often gain access to fisheries through multi-level bribery, from ministers to enforcement agents (108), and unfair bilateral fisheries agreements (21, 112, 113) biased toward the “wealthier and politically more powerful” home states of fishing companies “consciously seeking to profit from fishing in the waters of often weaker states” (80, 114). Beyond lobbying and corruption, large-scale fishing operations often benefit from heavy subsidies that lower costs (115), multiple vessels that spread risk, at-sea refueling and transshipment that evade monitoring, and access to legal markets that enable laundering.

Companies also bypass regulatory entry barriers and compliance costs by registering their vessels under FoC and landing their catch in low-risk ports with minimal fees and compliance requirements (116). Reflagging vessels with these states creates significant financial and operational advantages like reduced taxes, insurance fees, safety, and labor costs (4). FoCs also allow vessels to evade regulations in international waters, as those registered under states that have not ratified agreements like UNCLOS and RFMOs are exempt from their conservation, pollution, and quota restrictions. Avoiding controls in ports is also common. Vessels registered in states with weak corruption controls are disproportionately linked to IUU fishing, infrequent visit in PMSA-ratified countries, shorter port stays, and labor abuses (10). IUU fishing vessels are more likely to dock at free ports or in countries with high levels of illegal fishing, corruption, and ineffective catch inspections (83). Ports with high vessel traffic, nearby high-seas transshipments by FoC-flagged carriers, Chinese affiliations, frequent foreign vessel entries, and low monitoring capacity are more likely to attract high-risk FoC-flagged carriers (85). The effectiveness of measures against IUU at port-level generally reflects state governance quality but not national income (54, 117).

## 6. Market Developments and Innovations

IUU fishing has seen many changes over the past 30 y because of globalization, regulations, technology advances, and ecosystem changes. The depletion of traditional fishing areas and/or more stringent regulations and enforcement in the EEZs and regional areas of major fishing countries have led illegal fishing operations to move to more remote areas including the high seas and the EEZs of developing countries with limited regulatory oversight (118).

The global seafood market has become much larger between 1996 and 2022, due to increased supplies from aquaculture (36 MMT to 127 MMT per year) (119). The level of effort to catch fish has increased till the late 2010s, small-scale fishing boats have become increasingly motorized (120), and vessels have ventured further away from their port, increasing costs. Although the price of wild fish increased by about 50% between 2001 and 2023 (69), rising costs and competition have incentivized illegal practices. The global estimated volume of unreported catch has come down since the mid-1990s (81), suggesting that the volume of IUU-caught fish may be on a faster decline than legally caught fish or that more illegal catch is fraudulently reported as legal to authorities. While this could reflect the growing effectiveness of trade-restrictive measures, such as the EU carding system (121), it should be noted that these measures mostly target developing countries. They could thereby advantage large integrated companies and DWF fleets escaping or better equipped to (fraudulently) address such controls, thereby “further marginalizing small-scale fishers from developing countries” (78).

Seafood markets have also had to adapt to environmental changes, including fish stock movements associated with climate change opening up regulatory gaps (122). Climate change can induce or reinforce IUU fishing through the degradation and redistribution of fish stocks and their consequences on food security and fisheries profitability, thus reinforcing—for small-scale fishing especially—a “feedback loop of poverty, depletion, food and economic insecurity, and violence” that synergistically combines with climate change effects (123).

Technologies have changed both for fishing and surveillance. Illegal fishing uses more sophisticated technologies, including sonar, GPS, ocean conditions satellite data, satellite phones, transshipment, and trackable fish aggregating devices (FADs) to locate, capture, and market fish more efficiently and to coordinate operations, evade enforcement, and launder fish more easily (124). Yet, governments and organizations have increasingly used satellite tracking, AI, drones, and DNA-analysis to track suspicious fishing and illicit trade. Vessels can be tracked through combining satellite-based observations from Automatic Identification Systems (AIS) or Vessel Monitoring Systems (VMS), optical and radar imaging, and electromagnetic field detection (including those deliberately disabling their locational AIS and VMS signals). AI has enabled large-scale analysis of vessel behavior, including fishing patterns, transshipments, historical routes, and landings (125). Instituting mandatory permanent AIS and VMS signals from industrial fishing vessels, along with data receivability in court, would allow more systematic and comprehensive monitoring, surveillance, and accountability. Electronic monitoring systems combining GPS, activity sensors, and on-board cameras are being tested and deployed on vessels to document effort and measure catch, including illegal bycatch, reducing the need for onboard observers (126, 127). The widespread use of cellphones has also allowed more reporting by local fishers to authorities and the media, though enforcement still relies on state capacity and integrity.

The legal landscape has also evolved as more countries seek to counter illegal fishing and seafood laundering. Some, like Australia, Indonesia, and Palau, have seized or destroyed vessels caught fishing illegally in their EEZs. Others have strengthened port and market access restrictions through measures like PMSA and traceability schemes like the EU’s mandatory full digital traceability set to begin in 2026. The World Trade Organization agreement on fisheries subsidies adopted in 2022 could also reduce support for IUU fishing vessels, but its implementation remains uncertain (128). RFMOs have increased efforts against IUU fishing through better data sharing and monitoring mechanisms, yet serious noncompliance persists (129). Some retailers and consumers are demanding more transparency in seafood supply chains, pressuring companies to avoid IUU sourcing. Enforcement is mostly focused on fisheries management offenses, and often disregards other types of offenses (74, 130). Industrial-scale fishing remains able to avoid detection through diversion tactics and fraudulent practices, the corruption of local authorities and/or diplomatic pressure from their home government (18, 60, 131–133). Major fishing countries, especially those with large distant waters fishing fleets, have been criticized for not doing enough to track beneficial owners and punish illegal fishing by their companies (55, 116). More effective monitoring, control and surveillance, interdiction measures, suspension of subsidies, insurance, oil bunkering, and transshipments can help reduce industrial-scale illegal fishing (116, 134), but some measures, such as seafood certification, exclusive marine conservation, and top–down militarized forms of enforcement at sea can hurt small-scale fishing communities already made more vulnerable by industrial-scale IUU fishing (22).

## 7. Research Prospects and Limitations

Current research on IUU fishing is uneven (geographically and thematically). While multifactorial, this is largely due to resource constraints (135), fragmented methodologies, and insufficient access to data (136). Global and regional assessments are relatively robust, but there remains a dearth of comparative analysis of local case studies enabling more fine-grained yet generalizable findings. The best work often results from academic work and ground-based research by international nongovernmental and local civil society organizations combining remote sensing data with in-depth field investigations.

Continued development of detection technologies, multisensor fusion, and integrated platform systems is revolutionizing maritime domain awareness and enhancing the precision and scalability of IUU fishing detection (137), offering all-weather detection for noncooperative vessels, spatiotemporal tracking, and interpretation of different fishing vessel behaviors (138). Machine learning also accelerates multisources intelligence analysis to identify companies, trading links, and beneficial owners, while DNA-analysis improves traceability.

Understanding behavioral drivers of noncompliance in fisheries is also key to improving anti-IUU fishing outcomes. Rights-based management systems, for instance, motivate compliance by aligning small-scale fisher’s traditional fishing rights, economic incentives, and sustainable practices (54). These systems encourage behaviors that increase the value of catch rather than its volume, fostering long-term sustainability and compliance. Expanding these models could help address IUU fishing at its root by recognizing rights and reducing incentives for illegal practices. Other efforts can include mainstreaming investigative methodologies, enhancing collaboration between key stakeholders, and increasing transparency in each of supply chains, corporate ownership, and consumer markets, as well as policy and governance reforms including the adoption and implementation of key agreements such as the PSMA and WTO on harmful subsidies (139–141). Evidence-based policies informed by advanced analytics (e.g., species movements, fishing seasonality) and localized case studies (e.g., fishing practices, compliance culture) can enable proactive and anticipatory approaches to enforcement (e.g., patrol deployment, inspection targets) through quantitative risk-based inspection frameworks (142). Policy interventions include ending FoC registration and IUU fishing subsidies, ensuring mandatory electronic vessel identification, harmonizing legislations and strengthening legal institutions, securing universal public disclosure of beneficial ownership, requiring seafood producers to disclose their IUU risk assessments and mitigation measures, and building greater political will to facilitate information exchange and implement domestic and international regulations (143).

## 8. Conclusion

Despite increased regulation and enforcement efforts, industrial-scale illegal fishing remains a pressing global issue. The global scale and distribution of illegal fishing markets reflect geopolitical imbalances, economic structures, and states’ capacities that disproportionately concentrate the ills of illegality in poorer coastal states and benefits in wealthier nations. The complex and often opaque character of seafood supply chains allows illegally caught fish to be laundered into global markets through fraudulent documentation, transshipment, and weak monitoring at ports. This opacity also facilitates links with illegal markets such as human trafficking, drug smuggling, and other organized crimes. While technological and legal advancements help address some of these issues, enforcement remains fragmented and inconsistent across regions (144). Strengthening multilateral cooperation, closing governance gaps, and increasing transparency within supply chains is critical for not only reducing illegal fishing but also ensuring global stock sustainability. Doing so will not only require improved regulatory enforcement but also economic and social policies that address the key drivers of illegal fishing.

## Supplementary Material

Appendix 01 (PDF)

## Data Availability

Previously published data in Sea Around Us database were used for this work (81). Other data are included in the article and/or *SI Appendix*.
