# Supplementary material for: Casting light on the workings of illegal fishing markets
Source: Proc Natl Acad Sci U S A. 2026 Aug 10;123(33):e2512081123. doi: 10.1073/pnas.2512081123 (PMC13486526; doi:10.1073/pnas.2512081123)
Supplement: Supplementary file 1 [file pnas.2512081123.sapp.pdf]

Casting light on the workings of illegal fishing markets

Philippe Le Billon, Zelda Ladefoged, U. Rashid Sumaila

PNAS (2026)

Corresponding author: Philippe Le Billon, Department of Geography, 1984 West Mall, Vancouver, BC, V6T 1Z2, Canada; +1.778.952.2663; philippe.lebillon@ubc.ca

Supplementary Information

Definition of main terms

Beneficial ownership: The true individual(s) who ultimately own or control a company, asset, or vessel, even if it is registered under another name or entity. Often used to obscure accountability in cases of illegal or unethical practices.

Domestication: The process by which a foreign fishing vessel is registered under the flag of a coastal state and allowed to fish in its waters, often through joint ventures, licensing, or reflagging.

Flags of convenience: A practice where shipowners register vessels in countries with lenient regulations, low taxes, and limited oversight, often to avoid stricter labor, safety, or environmental laws in their home countries.

Forced labor: Work performed under coercion, threat, or deception, where individuals cannot freely leave their jobs. In fisheries, this often involves human trafficking, debt bondage, or abusive conditions on vessels.

Illegal fishing: Fishing activities that violate national, regional, or international laws or regulations. For example, fishing without a license, in prohibited areas, or during closed seasons.

Illicit fish trade: The unauthorized or illegal trade of fish and seafood products, including smuggling, mislabeling, and evading tariffs or regulations. It often involves fish caught through IUU practices.

Imported seafood products: While the 'nationality' of the catch is attached to the flag of the fishing vessel, imported seafood refers here to seafood caught in foreign EEZ or international

waters. Some variations may exist among national-level reporting systems. Note also that some imported seafood is processed and then re-exported to a third country.

IUU fishing: Stands for *Illegal, Unreported, and Unregulated* fishing. It includes illegal fishing, as well as fishing that is not reported or poorly reported to relevant authorities, and fishing in areas without effective governance or oversight.

Overfishing: The practice of harvesting fish at a rate that exceeds the species' ability to reproduce and replenish, leading to population decline and ecosystem imbalance.

Piracy: Criminal acts committed at sea, such as robbery, hijacking, or violence against ships, their crews, or cargo, typically for private gain and outside the authority of any state.

Seafood laundering: The process of disguising illegally caught fish as legal through methods such as false documentation, mixing legal and illegal catches, or rerouting through countries with weaker regulations.

Definition of industrial fishing

We follow the definition of the *Sea Around Us*, with the industrial fishing sector "consisting of relatively large motorized vessels, requiring large sums for their construction, maintenance and operation, either domestically, in the waters of other countries and/or the high seas, and landing a catch that is overwhelmingly sold commercially (as opposed to being consumed and/or given away by the crew). All gears that are dragged or towed across the seafloor or intensively through the water column using engine power (e.g., bottom- and mid-water trawls), no matter the size of the vessel deploying the gear are here considered industrial, … as are large pirogues … and 'baby trawlers' … capable of long-distance fishing", beyond depth greater than 200 meters or 50 km from inhabited coastal areas, whichever comes first (1). Whereas the industrial sector is large-scale and commercial in nature, the artisanal sector is small-scale and commercial, and the subsistence and recreational sectors are small-scale and non-commercial.

Estimation of the global marine fishing vessels

The FAO estimates the size of the global fishing fleet for 2022 at 4.9 million vessels (2). Information on length was only available for 45% of the global feet, but using a proportionality

rule, vessels under 12 meters would number 4.3 million (87%), 12-24 meters about 0.5 million (10%), and above 24 meters about 0.15 million (3%). The figure for vessels above 24 meters may be an overestimate as non-reporting countries are less likely to have vessels of that length. The International Maritime Organization (IMO) reports a figure of 64,000 vessels at or above 24 meters (3), a likely underestimate as not all fishing vessels of that length are registered with the IMO. About 81% of vessels with known zone of operation were operating in marine areas and 4% in marine and inland waters, suggesting a total figure of about 4.2 million vessels (2). About 71% of the global fishing fleet is in Asia (79% if considering only motorized vessels). A study by Rousseau, Watson, Blanchard and Fulton (4) estimated the number of unpowered vessels at 1.16 million, artisanal powered vessels at 2.11 million, and industrial powered vessels at 0.44 million in 2015, with a total of 3.7 million vessels, noting that the categories artisanal and industrial were determined by each country's own definitions or that of a geographically close country.

Estimation of illicit trade

The illicit trade is based on Sumaila et al.'s methodology (5) as the assumed fractions of the unreported catches estimated in the total reconstructed catch data (2015-2019) of the Sea Around Us database (1); noting that illicit trade is understood as trade dealing in illegally-caught seafood, for a discussion see (6). The low and high estimates are based on 50% and 80% of unreported industrial landed catches and 10% and 50% of artisanal landed catches being illicit trade, respectively. The estimated share of illicit trade originating from industrial fishing is 82-93% in volume and 74-90% in value, that of artisanal fishing is 7-18% in volume and 10-26% in value, out of the 8.4-15.4 MMT global annual illicit seafood trade valued at US$6.2-12.2 billion. These figures for annual illicit seafood trade are likely underestimated as a share of the reported marine catches is illegally-caught and the illegal catch from subsistence and recreational fisheries not accounted here. For a review of the methodologies used by the main studies estimating illegal fishing activities, see Macfadyen, Caillart and Agnew (7).

Harms caused by illegal fishing

Illegal fishing's *primary harm* is its negative impact on the health of targeted fish populations and associated effects on fisheries, livelihoods, food security, and fishing revenues. IUU fishing that exceeds quotas can lead to overfishing and often involves illegal methods, such as industrial trawling in zones reserved for artisanal fisheries. This undermines local livelihoods and food security, while the illegal fishing of high-value species can undermine public revenues. More broadly, illegal fishing can contribute to the degradation of marine habitats through harmful gear and practices, such as trawling in nursery areas, with long-term multi-species damages (8). Compared to legal operations, illegal fishing will often ignore catch limits and seasonal closures, target protected species, use destructive gear in sensitive areas (e.g. coral reefs), and evade monitoring, control, and surveillance. IUU's impacts are also felt more broadly within the seafood industry and society at large. For example, coastal communities' food security is undermined when illegally caught fish are exported to distant markets or laundered through local fishmeal factories, as in Gambia and Senegal (9, 10), unreported catch weakens fisheries management models by distorting stock assessments leading to unintended legal overfishing, and supply chain disruption exposes consumers to preventable health risks from poorly conserved illegally-caught fish or mislabeled products (8). The net global economic cost of IUU is difficult to assess, in part because estimating IUU catch is challenging (see SI) and illegally-caught seafood still largely ends up in economic circuits. Yet, the global annual economic value of products diverted from legitimate trade is estimated at $26-50 billion, with tax revenue losses around $2-4 billion for IUU-affected governments (mostly in Asia and Africa, each about $0.8– 1.4 billion), and $6.8 – 11.9 billion in potential losses for households (again mostly in Asia, $2.7 – 4.8 billion, and Africa, $1.8 – 3.1 billion) (5).

Illegal fishing also produces significant *secondary harms*. Fishing is a dangerous occupation, particularly on IUU vessels that fly flags of convenience with minimal regulation and no enforcement. These vessels often fail to meet international safety standards, operate beyond legal oversight, evade inspections and labor protections (including by remaining at sea for extended periods), and obscure beneficial ownership to avoid liabilities (11, 12). Violence sparked by competition over fishing rarely occurs at a scale and intensity seen in illegal markets

such as drug trafficking, but it frequently involves gear destruction, boat ramming, rock throwing, and more rarely fatal shootings (13, 14), particularly between small-scale and industrial vessels and in disputed and border areas (15, 16). Besides intentional attacks, the reckless actions of industrial vessels illegally operating in exclusive artisanal fishing zones may contribute up to 250 deaths per year amongst small-scale fishers operating pirogues in West Africa (17). While harm to marine animals is an inherent part of fishing, IUU practices are often especially destructive and inhumane (e.g. discarding live sharks after finning). Fisheries observers tasked with monitoring and reporting on fishing practices and catch are often the target of corruption, intimidation, and outright physical harm by IUU fishing captains or crews (18-20), with the privatization of the observation sector driving increased labor competition and reducing their protection (21). At-sea observers working alone are particularly vulnerable and between 2009 and 2023, at least 14 out of approximately 2,500 observers worldwide died under suspicious circumstances (21, 22). Violence associated with piracy targeting fishing vessels occasionally has its roots in illegal fishing practices by foreign vessels, as seen in Nigeria and Somalia where industrial-scale foreign fishing vessels threatened local fishing livelihoods and initiated armed responses that evolved into piracy (23-25), but see (26). Yet cracking-down on IUU fishing by *local* fishers can itself spur piracy, as observed in Indonesia (27).

Tertiary harms often involve connections with other illegal markets and organized crime, including illicit arms trade, drug trafficking, and smuggling of migrants (28-30). Industrial-scale fishing businesses are generally not owned or managed by criminal organizations with broader interests into other illegal markets (e.g. drug or arms trafficking), although this occurs in some high value and low volume fisheries such as abalone in South Africa, sturgeon fisheries for caviar in Russia, or Totoaba bladders in Mexico (31, 32). Some illegal fishing sectors have also been described as having mafia-like characteristics, such as illegal octopus fishing in Morocco (33) or the laundering of illegally caught bluefin tuna through tuna-fattening farms in the Mediterranean (34, 35). The involvement of fishers or their vessels in other illegal markets is not always voluntary nor willing. Drug cartels buy, steal or hijack fishing vessels and fishers are often deceived or coerced into facilitating transporting drugs or smuggled people (30, 36). For

example, small fishing vessels equipped with additional outboard engines (or '*lanchas*') are commonly used on smuggling routes between Latin America and the US (36, 37). Records of 1,764 cocaine seizures out of Colombia between 2016 and 2022 show that three quarters took place on such small vessels, dividing seizure risks (38). Larger, slower fishing vessels are also used, particularly for smuggling lower-value-per-volume drugs like cannabis between Morocco and Europe, while frozen seafood containers are commonly used for long-distance routes (29). Smugglers tend to prefer using 'clean vessels' and refrain from illegal fishing during operations to avoid heightened scrutiny (39). Despite these situational overlaps, the relationship between illegal fishing and drug trafficking is complex, and in the absence of conclusive, replicable evidence of their co-constitutive nature, no conclusions about their connection has been widely accepted within the literature (37).

IUU and abuses against crew members

Globally, marine fisheries employ about 16 million people, including 11.5 million in Asia, 2.2 million in Africa, 1.5 million in Latin America and the Caribbean, and 0.5 million in Europe, North America and Oceania, the vast majority being subsistence and artisanal fishers (2). Deaths and serious accidents among fishers are common (11), with some estimates being as high as 100,000 lives lost annually across the global fishing sector (40). Unsafe working conditions and human trafficking in the form of forced labor are deemed more frequent among IUU vessels, while abuses on distant-water fishing vessels in general are widespread (41-43), involving deceit, physical or psychological coercion, and movement restrictions (44). Uneven power dynamics mean that owners and captains often impose illegal practices on crew, with notably catch share systems incentivizing participation in illegal activities and threats of not getting paid if the catch is low (45). Crew members aboard industrial fishing vessels are generally recruited by labor intermediaries and hiring agencies, either locally if labor costs are low or among migrants from poorer countries like Cambodia, Indonesia and Myanmar (42, 46). Widespread abuses involved in human trafficking, including deceptive recruitment, wage theft, unsafe and exploitative working conditions, and abandonment when caught have been extensively documented (12, 41, 43). Global satellite data on fishing vessel activity estimates

that 57,000 to 100,000 crew members work under high risk of labor abuse aboard longliners and squid jiggers, most of which are flagged to Taiwan, China, Japan, and South Korea (47).

Crew members are mostly men between 25 and 45 years old with limited livelihood alternatives and formal education (48, 49). While forced labor among Southeast Asian migrants has received the most attention, these abuses are widespread, affecting workers from Europe, Russia, the US, UK, West Africa and the Pacific (48, 49). Migrant crew members are often undocumented or given false identity (39), with recruiters providing counterfeited documents including training certificates, police records, and Seafarer identification documents (e.g. forged Seaman Book). In sophisticated cases, computer hacking allows for the insertion of fraudulent data (50). Though this practice saves operators time and money, it leaves workers vulnerable to intensified abuse and with little legal or social recourse against human trafficking. Abuse can be reduced by requiring seafood companies to incorporate labor safety into their traceability systems, following international standards like the Work in Fishing Convention ILO 188, in effect since 2017 (51). When a vessel is caught, its owners will often make more efforts to repatriate a captain than the crew, who can linger in detention (e.g. 1-5 years) despite much lower - if any - responsibility (45). While captains are ultimately responsible for the safety of the crew and fishing practices, beneficial owners and local agents also have responsibilities towards their crews.

Enforcement challenges

Despite regional variations, international enforcement against IUU fishing remains weak due to gaps in participation, enforcement and coordination (52). First, some agreements have few signatories or long lack key parties (e.g. CTA), while others suffer from slow or inconsistent implementation (e.g. UNCLOS, UNFSA, or PSMA with regard to domestic vessels). Second, Flag states, particularly flags of convenience, often fail to enforce regulations, shifting the burden of monitoring and enforcement to coastal states and RFMOs (53). This leaves a major gap when coastal states lack monitoring, control and surveillance (MCS) and enforcement capacity, or when RFMOs, constrained by consensus-based decision-making, tolerate overfishing and

struggle to impose penalties (even on members). Third, the voluntary character of the UNFSA and IPOA-IUU further weakens their impact, as many states lack the capacity to implement regulations effectively (54). Finally, the anti-IUU regime generally suffers from fragmented legal frameworks, limited coordination between actors and instruments, and political obstacles including lack of will, corruption, and patchy implementation for lack of sufficient financial support and technical capacities (53, 55).

Enforcement primarily falls to Flag and coastal states (who use a combination of administrative and criminal sanctions) (56), along with RFMOs (which regulate by area by species), port states (where catch is landed), and importing states (57). Some states, like China, have tended to enforce strict regulations on domestic fishing but apply far weaker oversight to their vessels operating in foreign EEZs or on the high seas (58). Others, in part because of PSMA's focus on foreign vessels, do the reverse, leading some companies to shift their vessels to domestic flags to avoid more thorough inspections (59). There can also be some differences between national policies and sub-national practices, with some China provinces boosting their DWF capacity despite demands from the central government to curb down the number of vessels (60). RFMOs play a key role in regulating fishing in the high seas, but their effectiveness is weakened by member states prioritizing their own interests (e.g. foreign aid, geopolitics), internal tensions that reduce cooperation, and the inability to regulate non-member vessels (61). Based on a review of 168 interventions against illegal fishing, enforcement strategies mostly focus on increasing the risk of detection through surveillance, tracing devices and inspections (51%); raising costs and operational difficulty by seizing vessels, denying port services, removing fuel subsidies, creating artificial reefs, and banning transshipment (24%); reducing illegal profits through seafood traceability, supply chain disruptions, and restricted market access (13%); and eliminating justifications by clarifying regulations and educating consumers (12%) (62).

Effective fisheries management is frequently undermined by corruption, fraud, tax evasion, and embezzlement (20, 63), with cases documented in licensing, access agreement negotiations, regulation enforcement, taxation, and revenue management (64). In Fiji and the Solomon

Islands, fisheries officials have been caught diverting licensing fees into personal bank accounts, receiving direct payments from foreign corporations for advantageous license conditions, and granting 'private' licenses that do not appear on government registries (65). In 2005, the Solomon Islands' Auditor-General's Report into the Department of Fisheries and Marine Resources uncovered nearly USD $4 million in losses from diversions, misappropriations, unpaid fishing licence fees, and offset licence fee income (65). Corrupt authorities, including demands for bribes or a share of the catch, undermine fishers' willingness to comply with regulations and further reinforces defiance, thus reinforcing IUU fishing and mutual distrust, as seen in Kenyan and South African small-scale fisheries (66, 67). Fishing and seafood companies, including Asian and Western transnationals, also take on an active role in corruption or ignore malpractices by suppliers to protect their brands, with many cases of bribery involved in gaining access to fishing licenses, favorable quotas, tax avoidance, and the laundering of IUU-caught seafood (68). Some companies also gain significant influence in local fisheries by leveraging the economic and diplomatic weight of their home country and colluding with local politicians (68).

When caught, some operators will sink their vessels to destroy evidence before at-port-inspection or abandon the impounded ships and opt to start fleets anew as IUU profits can exceed the cost of recovering vessels and profits from a single fishing campaign can offset the cost of a used vessel (69). In terms of enforcement, regulatory agencies bear substantial costs for monitoring vast ocean areas, seizing vessels, and repatriating crews, while achieving limited meaningful deterrence. Vessel destruction or repurposing (e.g. as patrol vessel) are often preferred options to impoundment occupying precious docking space or crowding ports. Yet, destruction can be legally challenging and diplomatic pressure is sometimes put on local authorities to release boats, avoid criminal charges, and allow the repatriation of captains and key officers, though less so for crew members who have a different nationality (39, 45). In a relatively rare case of broad enforcement, Tanzania's High Court sentenced the Taiwanese captain of a Malaysian-flagged long-liner as well as its owner and the local agent, who was not officially registered, to twenty years in jail or a fine of $435,000 USD for unlawful possession of shark fins (70). The Indonesian crew alleged the captain withheld food if no fish was caught and

regularly threatened them with a gun to work harder (70). Penalties often fail to distinguish between industrial and artisanal fishers, resulting in disproportionately heavy burdens on the latter (e.g. up to 11 year imprisonment and 43,700 times the monthly minimal wage in Colombia). Penalties are particularly severe when IUU fishing threatens access to major seafood markets, as seen in Thailand and Vietnam's efforts to maintain access to the European Union market (71, 72).

In addition to the aforementioned lack of differentiation in penalty strength on the basis of operational scale, enforcement is often disproportionately biased against the small-scale fishing sector because it is easier to monitor (given its coastal character) and lacks the political protection enjoyed by the industrial sector (73). While this often ends up criminalizing and further marginalizing small scale fishers, it can also create scope for local guardianship efforts, such as indigenous initiatives (e.g. Indigenous Leadership Initiative (74)) and cooperative surveillance efforts (e.g. Senegalese CLPAs). In addition (and as a particularity of the fisheries industry), several non-governmental organizations (NGOs), such as Global Fishing Watch, Greenpeace, Sea Shepherds, and Environmental Justice Foundation, have taken on a growing role in monitoring IUU fishing, collecting data, and reporting violations. The Joint Analytical Cell (JAC) brings together the International MCS Network, TMT, Global Fishing Watch, C4ADS, and Skylight to facilitate data sharing against IUU fishing among state and non-state actors (75). While artificial intelligence and satellite-based technologies have improved the detection of IUU fishing (76, 77), enforcement often fails to keep pace.

References

1. D. Pauly, D. Zeller (2015) Catch Reconstruction: concepts, methods and data sources. in *Sea Around Us* (Sea Around Us, University of British Columbia).
2. Food and Agriculture Organization, *The State of World Fisheries and Aquaculture 2024* (FAO, 2024).
3. O. International Maritime, Enhancing fishing vessel safety to save lives.

4. Y. Rousseau, R. A. Watson, J. L. Blanchard, E. A. Fulton, Evolution of global marine fishing fleets and the response of fished resources. *Proceedings of the National Academy of Sciences* **116**, 12238–12243 (2019).

5. U. R. Sumaila *et al.*, Illicit trade in marine fish catch and its effects on ecosystems and people worldwide. *Science Advances* **6**, eaaz3801 (2020).

6. D. Belhabib, Response to Sumaila et al.'s Illicit trade in marine fish catch and its effects on ecosystems and people worldwide. *Science Advances* **6** (2020).

7. G. Macfadyen, B. Caillart, D. Agnew (2016) Review of studies estimating levels of IUU fishing and the methodologies utilized. (Poseidon Aquatic Resource Management Ltd.).

8. D. D. Miller, U. R. Sumaila, "Chapter 4 - IUU Fishing and Impact on the Seafood Industry" in Seafood Authenticity and Traceability, A. M. Naaum, R. H. Hanner, Eds. (Academic Press, San Diego, 2016), pp. 83–95.

9. Y. Loaiza, Illegal fishing for fish meal widespread in Ecuador marine reserve. *Mongabay Environmental News* (2023).

10. P. Majluf, K. Matthews, D. Pauly, D. J. Skerritt, M. L. D. Palomares, A review of the global use of fishmeal and fish oil and the Fish In:Fish Out metric. *Science Advances* **10**, eadn5650 (2024).

11. S. Chantavanich, W. Jitpong, Precarity and social protection of migrant fishers in Thailand: Case studies of death and disappearance at sea. *Marine Policy* **155**, 105743 (2023).

12. Environmental Justice Foundation (2022) The Ever-Widening Net. Mapping the scale, nature and corporate structures of illegal, unreported and unregulated fishing by the Chinese distant-water fleet. (Environmental Justice Foundation).

13. I. Okafor-Yarwood, The Cyclical Nature of Maritime Security Threats: Illegal, Unreported, and Unregulated Fishing as a Threat to Human and National Security in the Gulf of Guinea. *African Security* **13**, 116–146 (2020).

14. J. Spijkers *et al.*, Global patterns of fisheries conflict: Forty years of data. *Global Environmental Change* **57**, 101921 (2019).

15. C. DuBois, C. Zografos, Conflicts at sea between artisanal and industrial fishers: Inter-sectoral interactions and dispute resolution in Senegal. *Marine Policy* **36**, 1211–1220 (2012).

16. K. L. Seto *et al.*, Evidence of spatial competition, over resource scarcity, as a primary driver of conflicts between small-scale and industrial fishers. *Ecology and Society* **28** (2023).

17. D. Belhabib *et al.*, Catching industrial fishing incursions into inshore waters of Africa from space. *Fish and Fisheries* **21**, 379–392 (2020).

18. C. H. Faunce, J. Smith, A. Kingham, D. Jaszka, Fisheries observers as enforcement assets: 21 Years of lessons from the North Pacific. *Marine Policy* **158**, 105868 (2023).

19. A. Standing, Corruption and Industrial Fishing in Africa. (2008).

20. U. R. Sumaila, J. Jacquet, A. Witter, "When bad gets worse: corruption and fisheries" in Corruption, Natural Resources and Development. (Edward Elgar Publishing, 2017), pp. 93–105.

21. E. L. Garcia, Fisheries observers: An overlooked vulnerability for crime and corruption within the global fishing industry. *Marine Policy* **161**, 106029 (2024).

22. Association for Professional Observers (Observer Deaths and Disappearances | Association for Professional Observers (APO). in https://www.apo-observers.org/.

23. S. M. Glaser, P. M. Roberts, K. J. Hurlburt, Foreign Illegal, Unreported, and Unregulated Fishing in Somali Waters Perpetuates Conflict. *Frontiers in Marine Science* **6** (2019).

24. S. M. Mitchell, C. J. Schmidt, Insecure fisheries: How illegal, unreported, and unregulated fishing affects piracy. *Conflict Management and Peace Science* **41**, 313–338 (2024).

25. U. R. Sumaila, M. Bawumia, Fisheries, ecosystem justice and piracy: A case study of Somalia. *Fisheries Research* **157**, 154–163 (2014).

26. S. Jesperson, R. Henriksen, Piracy as a result of IUU fishing: Challenging the causal link. *African Security Review* **31**, 245–260 (2022).

27. A. Phayal *et al.*, All maritime crimes are local: Understanding the causal link between illegal fishing and maritime piracy. *Political Geography* **109**, 103069 (2024).

28. C. Haenlein (2017) Below the Surface How Illegal, Unreported and Unregulated Fishing Threatens our Security. in *RUSI Occasional Paper* (RUSI, London).

29. D. Belhabib, P. Le Billon, D. J. Wrathall, Narco-Fish: Global fisheries and drug trafficking. *Fish and Fisheries* **21**, 992–1007 (2020).

30. M. Mackay, B. D. Hardesty, C. Wilcox, The Intersection Between Illegal Fishing, Crimes at Sea, and Social Well-Being. *Frontiers in Marine Science* **7** (2020).

31. E. de Coning (2011) Transnational Organized Crime in the Fishing Industry. (UNODC).

32. E. Witbooi *et al.*, Organized crime in the fisheries sector threatens a sustainable ocean economy. *Nature* **588**, 48–56 (2020).

33. Seafood Source (2022) Moroccan octopus mafia accused of IUU fishing resulting in 60 percent population decline.

34. S. Adolf, *Tuna Wars: Powers Around the Fish We Love to Conserve* (Springer International Publishing, Cham, 2019).

35. U. R. Sumaila, L. Huang, Managing Bluefin Tuna in the Mediterranean Sea. *Marine Policy* **36**, 502–511 (2012).

36. M. P. Atkinson, M. Kress, R. Szechtman, Maritime transportation of illegal drugs from South America. *International Journal of Drug Policy* **39**, 43–51 (2017).

37. H.-T. Chiang, N. D. Cohen, S. Das, M. Sosnowski, G. A. Petrossian, An Empirical Assessment of Drug Trafficking Trends from Latin America into the United States: The Role of Fishing Vessels. *Deviant Behavior* **0**, 1–15 (2024).

38. Organized Crime and Corruption Reporting Project, Fishing Boats and Cargo Ships: How Colombian Cocaine Travels the World. *OCCRP* (2022).

39. I. Chapsos, S. Hamilton, Illegal fishing and fisheries crime as a transnational organized crime in Indonesia. *Trends Organ Crim* **22**, 255–273 (2019).

40. S. Willis, E. Holliday (2022) Triggering Death: Quantifying the True Human Cost of Global Fishing. (FISH Safety Foundation).

41. M. MacDonnell, P. Vandergeest, Fisher experience on distant water fishing vessels: the implications of variation by vessel type for employment standards. *Maritime Studies* **23**, 24 (2024).

42. M. Marschke, P. Vandergeest, Slavery scandals: Unpacking labour challenges and policy responses within the off-shore fisheries sector. *Marine Policy* **68**, 39–46 (2016).

43. E. R. Selig *et al.*, Revealing global risks of labor abuse and illegal, unreported, and unregulated fishing. *Nature communications* **13**, 1612 (2022).

44. B. D. Ratner, B. Åsgård, E. H. Allison, Fishing for justice: Human rights, development, and fisheries sector reform. *Global Environmental Change* **27**, 120–130 (2014).

45. G. Alonso, M. Marschke, Blue boats in deep waters: how aspects of IUU policy impact Vietnamese fish workers. *MAST. Maritime studies/Maritime studies* **22** (2023).

46. A. Kadfak, Labour in Fisheries Through Migration Studies: Burmese Fish Worker Regularisation and Agency in the Thai Fishing Industry. *Geopolitics* 10.1080/14650045.2024.2302422, 1–24 (2024).

47. G. G. McDonald *et al.*, Satellites can reveal global extent of forced labor in the world's fishing fleet. *Proceedings of the National Academy of Sciences* **118**, e2016238117 (2021).

48. A. Jaleel, H. D. Smith, The plight of the fishers and the management of IUU fishing. *Marine Policy* **150**, 105557 (2023).

49. S. Yea, C. Stringer, The informalisation of precarious work in fishing crew: Experiences of Fijian fishers on distant water vessels. *Marine Policy* **155**, 105709 (2023).

50. M. F, Links between Abuse of Seafarers' Documents with Trafficking in Human Beings, and Illegal Immigration in Southeast Asia. (2024).

51. K. Nakamura *et al.*, Seeing slavery in seafood supply chains. *Science Advances* **4**, e1701833 (2018).

52. M. S. Islam, A. S. M. M. Hasan, International legal arrangements for illegal, unreported, and unregulated fishing: legal mechanisms and challenges in Bangladesh. *Mar Dev* **2**, 28 (2024).

53. D. G. M. Miller, N. M. Slicer, E. Sabourenkov, An action framework to address Illegal, Unreported and Unregulated (IUU) fishing. *Australian Journal of Maritime & Ocean Affairs* **6**, 70–88 (2014).

54. M. Rosello, Cooperation and unregulated fishing: interactions between customary international law, and the European Union IUU fishing regulation. *Marine Policy* **84**, 306–312 (2017).

55. J. Lindley, E. J. Techera, Overcoming complexity in illegal, unregulated and unreported fishing to achieve effective regulatory pluralism. *Marine Policy* **81**, 71–79 (2017).

56. B. Kuemlangan *et al.*, Enforcement approaches against illegal fishing in national fisheries legislation. *Marine Policy* **149**, 105514 (2023).

57. G. Macfadyen, G. Hosch (2023) The Illlegal Unreported and Unregulated Fishing Risk Index. (Poseidon Aquatic Resource Management and Global Initiative Against Transnational Organized Crime).

58. H. Shen, S. Huang, China's policies and practice on combatting IUU in distant water fisheries. *Aquaculture and Fisheries* **6**, 27–34 (2021).

59. E. R. Selig *et al.*, Leveraging port state measures to combat illegal, unreported, and unregulated fishing. *Science advances* **11**, eads1592 (2025).

60. H. Zhou, Provincial variations and entrepreneurialism in the development of China’s Distant Water Fisheries (2011–2020). *Marine Policy* **147**, 105344 (2023).

61. H. Sinan, M. Bailey, W. Swartz, Disentangling politics in the Indian Ocean Tuna Commission. *Marine Policy* **133**, 104781 (2021).

62. N. Marteache, M. C. Sosnowski, G. A. Petrossian, "A Review of Responses to IUU Fishing around the World through the Lens of Situational Crime Prevention" in The Emerald Handbook of Crime, Justice and Sustainable Development*,* J. Blaustein, K. Fitz-Gibbon, N. W. Pino, R. White, Eds. (Emerald Publishing Limited, 2020), pp. 485–512.

63. A. Standing, Corruption and state-corporate crime in fisheries. (2015).

64. Y. Yan, A. Graycar, Exploring corruption in fisheries. *Natural Resources Forum* **44**, 176–190 (2020).

65. Q. Hanich, M. Tsamenyi, Managing fisheries and corruption in the Pacific Islands region. *Marine Policy* **33**, 386–392 (2009).

66. F. Nunan *et al.*, Compliance, corruption and co-management: how corruption fuels illegalities and undermines the legitimacy of fisheries co-management. *International Journal of the Commons* **12**, 58–79 (2018).

67. A. Sundström, Corruption in the commons: Why bribery hampers enforcement of environmental regulations in South African fisheries. *International Journal of the Commons* **7**, 454–472 (2013).

68. D. Belhabib, P. Le Billon, Fish crimes in the global oceans. *Science Advances* **8**, eabj1927 (2022).

69. U. R. Sumaila, J. Alder, H. Keith, Global scope and economics of illegal fishing. *Marine Policy* **30**, 696–703 (2006).

70. Sea Shepherd (2018) Taiwanese Captain Sentenced 20 Years for Shark Finning in Tanzania. in *Sea Shepherd Global*.

71. D. E. Kim, S. S. Lim, Economic impacts of the European Union carding system on global fish trade. *Marine Policy* **165**, 106208 (2024).

72. T. Vinh, C. Chi (2024) 4 Vietnamese fishers jailed over illegal exits, IUU fishing. in *Tuoi Tre News*.

73. I. Okafor-Yarwood, N. I. Kadagi, D. Belhabib, E. H. Allison, Survival of the Richest, not the Fittest: How attempts to improve governance impact African small-scale marine fisheries. *Marine Policy* **135**, 104847 (2022).

74. Indigenous Leadership Initiative (Indigenous Guardians. in *Indigenous Leadership Initiative*.

75. Joint Analytical Cell (2023) Data, Intelligence and Tools to Combat Illegal Fishing. (Joint Analytical Cell).

76. H. Welch *et al.*, Harnessing AI to map global fishing vessel activity. *One Earth* **7**, 1685–1691 (2024).

77. K. Zuzanna, U. Tomasz, G. Michał, P. Robert, How High-Tech Solutions Support the Fight Against IUU and Ghost Fishing: A Review of Innovative Approaches, Methods, and Trends. *IEEE Access* **10**, 112539–112554 (2022).
